# Nanobody-Alkaline Phosphatase Fusion Protein-Based Enzyme-Linked Immunosorbent Assay for One-Step Detection of Ochratoxin A in Rice

**DOI:** 10.3390/s18114044

**Published:** 2018-11-20

**Authors:** Zhichang Sun, Xuerou Wang, Qi Chen, Yonghuan Yun, Zongwen Tang, Xing Liu

**Affiliations:** College of Food Science and Technology, Hainan University, 58 Renmin Avenue, Haikou 570228, China; 13919003678@163.com (Z.S.); w490487@126.com (X.W.); qichen@hainu.edu.cn (Q.C.); yunyonghuan@foxmail.com (Y.Y.); tzwcx@163.com (Z.T.)

**Keywords:** mycotoxin, nanobody, immunoassay, alkaline phosphatase

## Abstract

Ochratoxin A (OTA) has become one a focus of public concern because of its multiple toxic effects and widespread contamination. To monitor OTA in rice, a sensitive, selective, and one-step enzyme-linked immunosorbent assay (ELISA) using a nanobody-alkaline phosphatase fusion protein (Nb28-AP) was developed. The Nb28-AP was produced by auto-induction expression and retained an intact antigen-binding capacity and enzymatic activity. It exhibited high thermal stability and organic solvent tolerance. Under the optimal conditions, the developed assay for OTA could be finished in 20 min with a half maximal inhibitory concentration of 0.57 ng mL^−1^ and a limit of detection of 0.059 ng mL^−1^, which was 1.1 times and 2.7 times lower than that of the unfused Nb28-based ELISA. The Nb28-AP exhibited a low cross-reactivity (CR) with ochratoxin B (0.92%) and ochratoxin C (6.2%), and an ignorable CR (<0.10%) with other mycotoxins. The developed Nb-AP-based one-step ELISA was validated and compared with a liquid chromatography-tandem mass spectrometry method. The results show the reliability of Nb-AP-based one-step ELISA for the detection of OTA in rice.

## 1. Introduction

Ochratoxin A (OTA), a secondary mold metabolite produced by *Aspergillus* and *Penicillium*, is one of the most frequently reported mycotoxins in various types of food such as cereal products, dried fruits, and coffee [1]. OTA can induce multiple toxic effects to humans and animals, and was categorized as group 2B (possible carcinogens to humans) by the International Agency for Research on Cancer [2,3,4]. A recent study has provided evidence for the mechanism of OTA carcinogenesis in humans [5]. Considering the toxicities of OTA, most countries have set regulations for it in different food products. The maximum level is 10 μg kg^−1^ in dried vine fruit, 5 μg kg^−1^ in unprocessed cereals, and 3 μg kg^−1^ in cereal products set by the European Union [6]. Therefore, adequate techniques for OTA screening in agricultural commodities are indispensable.

Numerous analytical techniques have been applied to detect OTA in food samples including chromatography, mass spectrometry, electrophoresis, and combined methods [7,8,9,10,11,12]. Nevertheless, these methods require sophisticated equipment, complicated sample preparation, and time-consuming detection. Alternatively, immunoassays are usually recognized as a rapid and high-throughput detection method. A traditional approach of screening, which is the enzyme-linked immunosorbent assay (ELISA), has been applied for high throughput screening of OTA and other analytes [13,14,15,16,17]. For the above ELISAs, enzymes are covalently linked to analytes or antibodies to catalyze signal generation. Nevertheless, the chemical conjugation methods yield randomly cross-linked heterogeneous products and may lead to activity reduction or loss of the enzyme and antibody [18].

Recent advances in genetic engineering have promoted the development of different derivatives of intact antibodies, such as single chain antibody fragments (scFvs), to produce novel immunodiagnostic reagents with lower cost and better stability than the intact antibody [19]. The construct of scFv-alkaline phosphatase (AP) genetic fusions can avoid the preparation of enzyme-antibody conjugates produced by chemical labeling method and shorten assay time compared with the standard ELISA method, in which the addition of a primary antibody and a secondary antibody labeled with enzyme molecules is performed in sequence [20]. However, low yield, poor solubility, and stability of scFvs limit their further applications, and additional genetically engineered modification is required [21]. The heavy chain antibody (HCAb), which naturally lacks light chains, was firstly found in camelids [22]. By cloning, expression, and purification of the heavy chain gene of HCAb (VHH), a kind of recombinant single domain antibody (nanobody, Nb) was obtained, and the Nb still retains the antigen-binding activity of HCAb [23]. The Nb possesses numerous beneficial properties, such as high solubility, high expression, and high tolerance to harsh conditions [23]. Thus, the above unique features of Nb make it an excellent alternative to scFv. The Nb is also very suitable for gene engineering due to its single domain nature. The Nb-AP fusions have been successfully produced and applied to the one-step detection of different targets, such as fumonisin B_1_ (FB_1_) and tetrabromobisphenol A [24,25].

In this work, the Nb-AP fusion protein (Nb28-AP) against OTA was produced by auto-induction expression, and characterized by thermal stability analysis and organic solvent tolerance evaluation. The purified Nb28-AP was applied to establish a sensitive, rapid, and selective ELISA for screening of OTA in rice. The Nb28-AP can serve as the primary antibody and reporter molecule, and thus combine the two steps of addition of a primary antibody and a secondary antibody labelled with enzyme in standard ELISA into one-step operation. This work proves that the Nb-AP is a sturdy and hopeful immunoreagent for the analysis of OTA and other low molecular weight analytes in food.

## 2. Materials and Methods

### 2.1. Reagent and Chemicals

Standards of OTA, ochratoxin C (OTC), and FB_1_ were obtained from Pribolab (Singapore). Standards of aflatoxin B_1_ (AFB_1_) and zearalenone (ZEN) were procured from Fermentek (Jerusalem, Israel). Ochratoxin B (OTB) was purchased from Bioaustralis (Smithfield, NSW, Australia). Non-fat milk powder, 96-well microplates, *p*-nitrophenyl phosphate (pNPP) substrate, and 3,3′,5,5′-teramethylbenzidine (TMB) were obtained from Sangon Biotech (Shanghai, China). HRP-labeled mouse anti-6 × His tag monoclonal antibody was procured from CWBIO (Beijing, China). Nickel-nitilotriacetic acid (Ni-NTA) sepharose was purchased from Solarbio (Beijing, China). Unfused Nb28 was prepared in the previous study [26]. All inorganic chemicals and organic solvents were analytical grade.

### 2.2. Expression, Purification, and Identification of the Nb28-AP

The auto-induction expression and purification of Nb28-AP was performed as described previously [27]. The size and purity of Nb28-AP was determined by sodium dodecyl sulfate-polyacrylamide gel electrophoresis (SDS-PAGE). To determine the AP enzymatic activity of Nb28-AP, a colorimetric analysis was performed as follows. Serial dilution of Nb28-AP (100 μL/well) and the equal volume of 3.8 mM pNPP substrate solution (pH 9.8) containing 1 M diethanolamine and 0.5 mM MgCl_2_ was mixed. After incubation at 37 °C for 5 min, the color reaction was stopped with 50 μL/well of 3 M sodium hydroxide solution. The absorbance at 405 nm was determined on a microplate reader. In addition, the reactivity of Nb28-AP with OTA was assessed using an indirect competitive Nb-ELISA as described previously with some modifications [26]. In brief, 50 μL of Nb28-AP solution (800 ng mL^−1^ in PBS) and 50 μL of OTA standard solution were added to the microtiter plate coated with 2 × 10^3^ ng mL^−1^ of OTA-BSA conjugate. After incubation for 30 min at 37 °C, the microplate was subjected to another incubation with 100 μL of HRP-labeled mouse anti-6 × His tag monoclonal antibody solution (200 ng mL^−1^ in PBS). The binding signal was detected after incubation with 100 μL of TMB substrate solution.

### 2.3. Nb-AP-Based One-Step ELISA for OTA

The Nb-based one-step ELISA was conducted as follows. In brief, a 96-well microplate was coated with 100 μL/well of OTA-BSA (500 ng mL^−1^ in PBS) at 4 °C overnight. After discarding the solutions, the plate was washed three times with PBS containing 0.05% Tween20 (PBST) and incubated with 300 μL/well of blocking agent (3% non-fat milk in PBS) at 37 °C for 1 h. After three more washes, 50 μL of OTA standard solutions with various concentrations as well as 50 μL of diluted Nb28-AP solution were added to the plate, followed by incubation at 37 °C for 45 min. The plate was rinsed three times with PBST, and 100 μL/well of pNPP substrate solution were added. The enzymatic reaction was immediately terminated with 50 μL/well of 3 M sodium hydroxide solution after incubation at 37 °C for 5 min. The absorbance wells at 405 nm was tested on a microplate reader, and the standard inhibition curve was established as described previously [26].

### 2.4. Selectivity of the Nb-AP-Based One-Step ELISA

To evaluate the selectivity of developed Nb-AP-based one-step ELISA for OTA, the cross-reactivity (CR) of Nb28-AP with structural analogs of OTA including OTB and OTC and several cereal mycotoxins (AFB_1_, ZEN, and FB_1_) was determined. The CR (%) was calculated according to the equation CR (%) = [IC_50_ (OTA)/IC_50_ (tested analytes)] × 100 [28], where IC_50_ refers to the half maximal inhibitory concentration.

### 2.5. Sample Preparation and Analysis

Rice samples for the recovery experiment were purchased from local markets and supermarkets in Haikou, China. OTA-contaminated rice samples were collected from markets in the United States. For the Nb-AP-based ELISA analysis, rice samples were pretreated as follows. Briefly, 1 g of ground rice sample was weighed and immersed with 2 mL of 50% methanol-PBS. After 15 min of vigorous shaking and ultrasonication extraction, the mixture was centrifuged (10,000 g, 10 min) to separate the supernatant containing OTA. The supernatant was directly diluted 1:4 with PBS for Nb-AP-based ELISA analysis. For the liquid chromatography tandem mass spectrometry (LC-MS/MS) analysis, the sample pretreatment and measurement was conducted according to the previous study [29].

## 3. Results and Discussion

### 3.1. Expression, Purification, and Identification of the Nb28-AP

**Figure 1:**
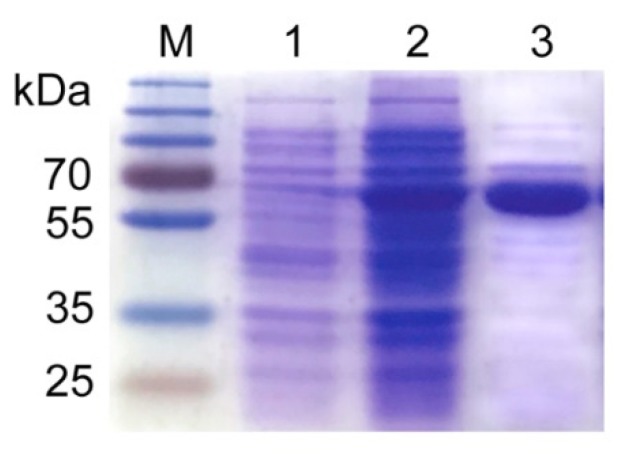
Characterization of Nb28-AP by SDS-PAGE. Lane M: Prestained protein ladder. Lane 1: Whole-cell protein before auto-induction. Lane 2: Whole-cell protein after auto-induction. Lane 3: Nickel sepharose column-purified Nb28-AP.

In this work, the anti-OTA nanobody Nb28 fused with alkaline phosphatase (Nb28-AP) served as the primary antibody and reporter molecule. Instead of chemical synthesis, the Nb28-AP was produced in a stable and sustainable way by protein engineering. The Nb28-AP was auto-induction expressed by the engineered *E. coli* BL21(DE3) strain and purified using a Ni-NTA sepharose-packed column. The prokaryotic expression and purification of Nb28-AP were characterized by SDS-PAGE (Figure 1). In contrast with the whole cell protein before induction (Lane 1), the target band at 65 kDa was isolated from the whole cell protein after induction (Lane 2). The result was in accordance with that of previously reported IPTG-induction method [29]. Therefore, these results indicated the successful expression of Nb28-AP using the auto-induction method. Moreover, the Nb28-AP with high purity was obtained by the Ni-NTA-based affinity purification chromatography (Lane 3). The AP enzymatic activity of Nb28-AP was tested by a colorimetric analysis (Figure S1A). The signal intensity at 405 nm increased sharply as the amount of Nb28-AP increased from 0.13 pmol to 0.43 pmol. The signal intensity approached the saturation point when the amount of Nb28-AP reached 4.2 pmol. Moreover, the reactivity of Nb28-AP with OTA was determined by an indirect competitive Nb-ELISA, and a standard inhibition curve was constructed (Figure S1B). As the OTA concentration increased, the binding between Nb28-AP and OTA-BSA decreased with an IC_50_ of 1.6 ng mL^−1^. These results indicated that the Nb28-AP retained acceptable enzymatic activity of AP and reactivity with OTA.

### 3.2. Characterization of the Nb28-AP

**Figure 2:**
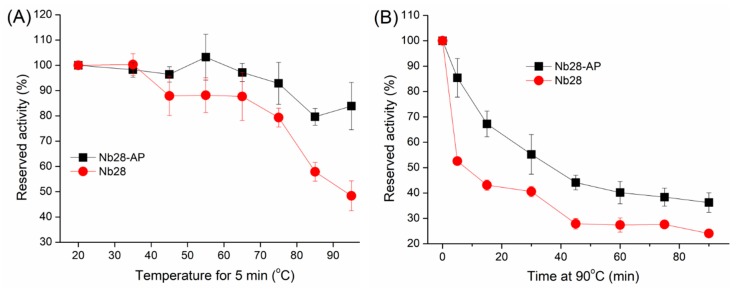
Thermal stability of Nb28 and Nb28-AP. Nb28 and Nb28-AP at working concontrations were incubated at serial temperatures (35, 45, 55, 65, 75, 85, 95 °C) for 5 min (**A**) or at 90 °C for various lengths of time (0, 5, 15, 30, 45, 60, 75 min) (**B**). After cooling to room temperature, the reserved activity of Nb28 and Nb28-AP was measured by an indirect ELISA. The error bars represent the standard deviation of three independent tests.

Nbs are robust under harsh conditions and resist chemical and thermal denaturation because of their unique structures, such as the intramolecular disulfide bonds in complementary determining regions [23]. In this work, evaluation of thermal stability and organic solvent tolerance was performed to characterize Nb28-AP (Figure 2 and Figure S2). The retained antigen binding activity was used for evaluation, which was calculated as [OD_405_ (antibody treated with heating or organic solvents)/OD_405_ (untreated antibody)] × 100. The thermal stability of Nb28-AP was characterized by comparison to that of the unfused Nb28 (Figure 2). After 5 min incubation at serial temperatures, the reserved activity of both Nb28 and Nb28-AP decreased as the temperature increased (Figure 2A). However, the Nb28-AP still retained 85% activity which was higher than that of Nb28 (50%) as the temperature reached 95 °C. To further illustrate the difference in thermal stability, the effect of incubation (90 °C) time on the activities of both antibodies were tested (Figure 2B). The activities of both antibodies decreased as the incubation time increased. Nonetheless, the Nb28-AP still exhibited a higher reserved activity (40%) than that of Nb28 even when the incubation time was up to 90 min. Thus the Nb28-AP has a better thermal stability than Nb28. To evaluate the organic solvent tolerance of Nb28-AP, the activity of Nb28-AP exposing in methanol, ethanol, acetonitrile, acetone, dimethyl sulfoxide (DMSO), and dimethyl formamide (DMF) with various concentrations (0–80%) was investigated (Figure S2). The binding activity ranged from 80% to 106% as the methanol concentration varied from 0% to 40%. But it decreased to 72% as the methanol concentration reached 80% (Figure S2A). Similar results were obtained from acetonitrile, DMSO, and DMF, except that the lower binding activity was observed as the concentraion of those solvents was 80% (Figure S2C,E,F). The binding activity declined significantly as the ethanol concentration increased from 0% to 80% except for 40% (Figure S2B). The Nb28-AP exhibited the highest tolerance to acetone among the tested organic solvents, and retained 118% of its binding activity at 80% of acetone (Figure S2D). Thus, the Nb28-AP was proved to be a robust bioreceptor with high thermal stability and good tolerance to organic solvents.

### 3.3. Nb-AP-Based one-Step ELISA for OTA

**Figure 3:**
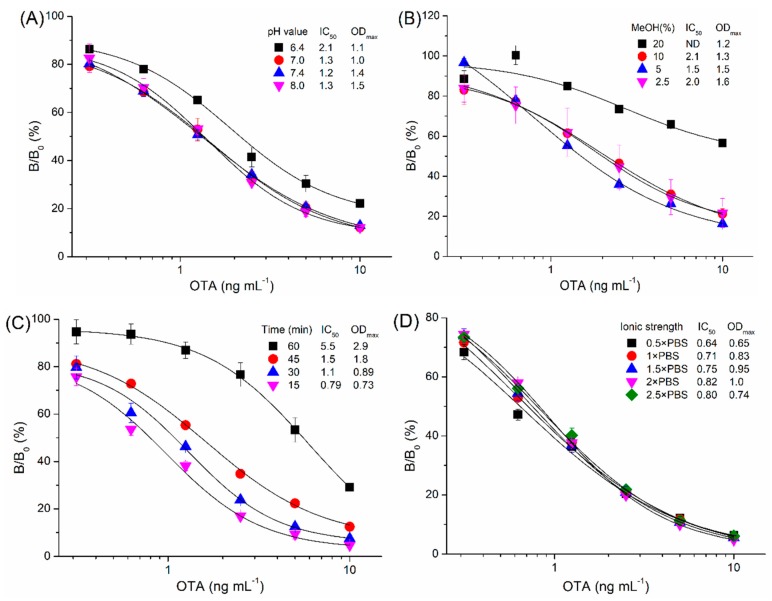
Effects of pH value (**A**), methanol (**B**), competitive time (**C**), and ionic strength (**D**) on the performance of Nb-AP-based one-step ELISA. The error bars represent the standard deviation of three independent tests.

**Figure 4:**
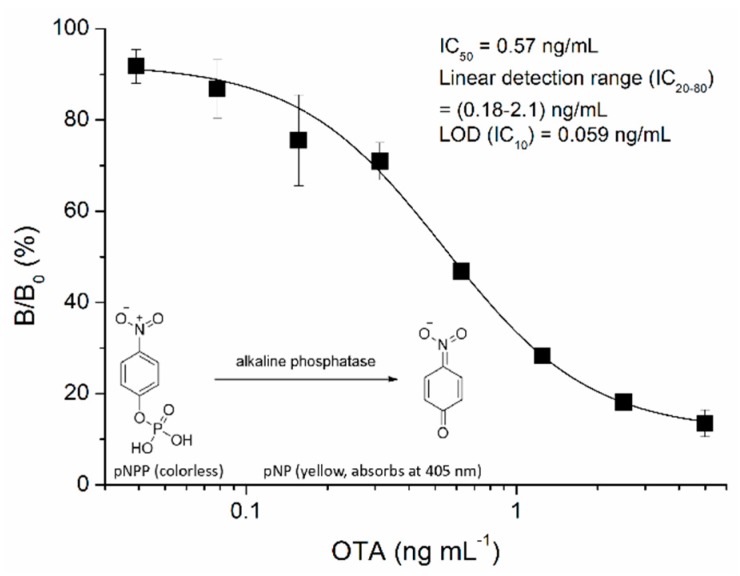
Standard direct competitive inhibition curve of Nb-AP-based one-step ELSIA for OTA under the optimized conditions. The error bars represent the standard deviation of five independent tests.

The optimal concentrations of OTA-BSA (500 ng mL^−1^ in PBS) and Nb28-AP (0.4 μg mL^−1^ in PBS) were first selected by a checkerboard titration [30]. In addition, the effects of various conditions on the assay performance were assessed, including pH, methanol, ionic strength, and the competitive time between the free OTA and the coating antigen for binding to Nb28-AP. One-step assays were performed with various pH values (Figure 3A). The maximum value of absorbance at 405 nm (OD_max_) decreased from 1.5 to 1.1 as the pH value decreased from 8.0 to 6.4. There was significant variation for the IC_50_ value when pH was less than 7.0, and a pH value of 7.4 was selected for the lowest IC_50_ value (1.2 ng mL^−1^). OTA is highly soluble in polar organic solvents, and methanol is usually selected as the solvent for OTA; however, it can interfere the recognition of the antibody to the antigen [31]. As shown in Figure 3B, the OD_max_ decreased from 1.64 to 1.22 as the final methanol concentration in the assay buffer increased from 2.5% to 20%. Since the lowest value of IC_50_ (1.5 ng mL^−1^) was obtained at 5% of final methanol concentration, the PBS buffer (pH 7.4) containing 10% methanol was selected for further research. To shorten the assay time, the competitive time between OTA and OTA-BSA for binding to Nb28-AP was optimized (Figure 3C). Both the IC_50_ value (from 0.79 to 5.5 ng mL^−1^) and OD_max_ (from 0.73 to 2.9) increased with the time increasing from 15 to 60 min, and 15 min was selected as the optimal competitive reaction time for the lowest value of IC_50_ (0.79 ng mL^−1^). The effects of assay buffer with different ionic strength on the assay performance are shown in Figure 3D. Both the IC_50_ value and OD_max_ slightly increased as the ionic strength did not exceed 2× PBS, and 0.5× PBS of ionic strength was selected for further research. On basis of the optimal working conditions, the standard direct competitive inhibition curve of one-step ELISA for OTA was established (Figure 4). The assay has a limit of detection (LOD = IC_10_, the 10% inhibitory concentration) of 0.059 ng mL^−1^, an IC_50_ of 0.57 ng mL^−1^ as well as a linear detection range (IC_20–80_) of 0.18–2.1 ng mL^−1^. Compared with other immunoassays for OTA, the Nb-AP-based one-step ELISA shows certain advantages in analytical performance, such as detection sensitivity and assay time [26,28,30,31,32]. As shown in Table 1, it shows the improvement of 1.1 times in IC_50_, 2.7 times in LOD, and 3.5 times in assay time compared with the Nb28-based ELISA in our previous study [26]. The LODs for chemiluminescent, fluorescent, and electrochemical methods are often lower, but they are limited for more time-consuming operation and the requirement of special equipment.

### 3.4. Selectivity of the Nb-AP-Based One-Step ELISA

The selectivity of Nb28-AP-based one-step ELISA for OTA was evaluated by comparing the IC_50_ value of OTA with that of OTB, OTC, AFB_1_, ZEN, and FB_1_ (Figure S3) in one-step assays. As shown in Table 2, the Nb28-AP exhibited a high specificity in recognition of OTA and a low CR with OTB (0.92%) and OTC (6.2%). Considering 10% of CR with OTB for the unfused Nb28, the lower CR with OTB for Nb28-AP may be attributed to the steric hindrance caused by fused AP [27]. The Nb28-AP shows ignorable CR (lower than 0.1%) with other mycotoxins, which was similar to Nb28 [26]. These results demonstrated the high selectivity of Nb-AP-based one-step ELISA for the detection of OTA.

### 3.5. Sample Analysis and Validation

To evaluate the effectiveness of Nb-AP-based one-step ELISA for OTA in sample analysis, rice samples were spiked with different contents of OTA (3, 6, 12, and 30 μg kg^−1^) and subjected to the spike-and-recovery study. The average intra-assay recoveries ranged from 70% to 103% and the relative standard deviation (RSD) ranged from 2.4% to 7.1% (Table 3). For inter-assay, the recovery and RSD was in a range of 70–107% and 1.4–6.2%, respectively (Table 3). Three OTA-contaminated rice samples that had been analyzed previously [26] were determined by the Nb-AP-based one-step ELISA and validated by LC-MS/MS (Figure S4). The contents of OTA (1.9, 2.1, and 2.2 μg kg^−1^) tested by the Nb-AP-based one-step ELISA matched well with those (1.6, 1.8, and 2.1 μg kg^−1^) tested by LC-MS/MS (Table 4). These results indicate the reliability of the developed Nb-AP-based one-step for OTA detection in rice samples with acceptable accuracy and precision.

## 4. Conclusions

Thus this study presented a one-step ELISA using the bifunctional Nb-AP for determining OTA in rice with high sensitivity and selectivity. Combining the high catalytic efficiency of AP and the advantages of Nb, the Nb-AP became a very promising bioreceptor. Compared to the monoclonal antibodies produced by hybridoma technology, the Nb-AP costs much less for development and production. Due to the homodimeric nature of AP, genetic attachment of the AP to Nb can enlarge the paratopes, enhance the flexibility and improve the affinity of fused Nb. As demonstrated here, the AP fusion technology can accelerate the detection procedure and reduce variability by eliminating a step. Moreover, it can minimize the CR and enhance the sensitivity of the procedure. Thus, the AP fusion technology is suitable for the analysis of OTA and other toxic compounds with low molecular weight.

## Figures and Tables

**Table 1:** Overview of recently reported immunoassays for ochratoxin A.

Materials Used	Method Applied	Assay Time	LOD	Reference
Nb-AP	ELISA	20 min	0.059 ng mL^−1^	this work
Nb	ELISA	70 min	0.16 ng mL^−1^	[26]
mAb, amine-functionalized magnetic nanoparticles	chemiluminescence immunoassay	60 min	1.39 pg mL^−1^	[33]
Nb-AP	fluorescence enzyme immunoassay	45 min	0.04 ng mL^−1^	[29]
mAb, gold nanoparticles	lateral flow immunoassay	10 min	10 ng mL^−1^	[32]
MAb, Cds NRs/FTO photoelectrode, G-quadruplex/hemin, Gold nanoflower, SiO_2_@Cu^2+^	photoelectrochemical immunosensor	340 min	0.02 pg mL^−1^	[34]

**Table 2:** Cross-reactivity of Nb28-AP with OTA structural analogs and other mycotoxins.

Analytes	IC_50_ (ng mL^−1^)	CR (%)
OTA	0.57	100
OTB	62	0.92
OTC	9.2	6.2
AB_1_	>1000	<0.10
ZEN	>1000	<0.10
FB_1_	>1000	<0.10

**Table 3:** Recoveries of OTA from the spiked rice samples by Nb-AP-based one-step ELISA.

Spiked OTA (μg kg^−1^)	Mean ± SD (μg kg^−1^)	Recovery (%)	RSD (%)
Intra assay (*n* = 3) ^a^			
3	3.1 ± 0.10	103	3.2
6	5.8 ± 0.41	97	7.1
12	11 ± 0.26	92	2.4
30	21 ± 1.1	70	5.2
Inter assay (*n* = 3) ^b^			
3	3.2 ± 0.20	107	6.2
6	5.9 ± 0.12	98	2.0
12	11 ± 0.16	92	1.4
30	21 ± 1.3	70	6.2

^a^ Each assay was conducted in three replicates on the same day. ^b^ The assays were conducted on three different days.

**Table 4:** Detection of OTA in rice samples by the Nb-AP-based one-step ELISA and LC-MS/MS.

Rice	Nb-AP-Based ELISA ^a^ Mean ± SD (μg kg^−1^)	RSD (%)	LC-MS/MS ^a^ Mean ± SD (μg kg^−1^)	RSD (%)
1	2.1 ± 0.13	6.2	1.8 ± 0.12	6.7
2	2.2 ± 0.16	7.3	2.1 ± 0.11	5.2
3	1.9 ± 0.12	6.3	1.6 ± 0.12	7.5

^a^ Each sample was determined in triplicate.

## Supplementary Materials

Supplementary Materials can be found at http://www.mdpi.com/1424-8220/18/11/4044/s1, Figure S1: AP enzymatic activity and anti-OTA reactivity analysis of Nb28-AP. Figure S2: The solvent tolerance of Nb28-AP for methanol (**A**), ethanol (**B**), acetonitrile (**C**), acetone (**D**), DMSO (**E**), and DMF (**F**). Figure S3: The chemical structures of OTA, OTB, OTC, AFB_1_, FB_1_, and ZEN. Figure S4: LC–MS/MS analysis of the OTA-contaminated rice sample (**A**) and the OTA standard (**B**).
