# Supplementary material for: Nanobody-Alkaline Phosphatase Fusion Protein-Based Enzyme-Linked Immunosorbent Assay for One-Step Detection of Ochratoxin A in Rice
Source: Sensors (Basel). 2018 Nov 20;18(11):4044. doi: 10.3390/s18114044 (PMC6263964; doi:10.3390/s18114044)
Supplement: Supplementary file 1 [file sensors-18-04044-s001.pdf]

Supplementary Information

Nanobody-Alkaline phosphatase Fusion Protein-Based Enzyme-Linked Immunosorbent Assay for One-Step Detection of Ochratoxin A in Rice

Zhichang Sun †, Xuerou Wang †, Qi Chen, Yonghuan Yun, Zongwen Tang and Xing Liu *

College of Food Science and Technology, Hainan University, 58 Renmin Avenue, Haikou 570228, China; 13919003678@163.com (Z.S.); w490487@126.com (X.W.); qichen@hainu.edu.cn (Q.C.); yunyonghuan@foxmail.com (Y.Y.); tzwcx@163.com (A.T.)

* Corresponding author: xliu@hainu.edu.cn; Tel.: +86-898-6619-3581

† These authors contributed equally to this work.

Table of contents

Figure S1 ..... S-1

Figure S2 ..... S-2

Figure S3 ..... S-3

Figure S4 ..... S-4

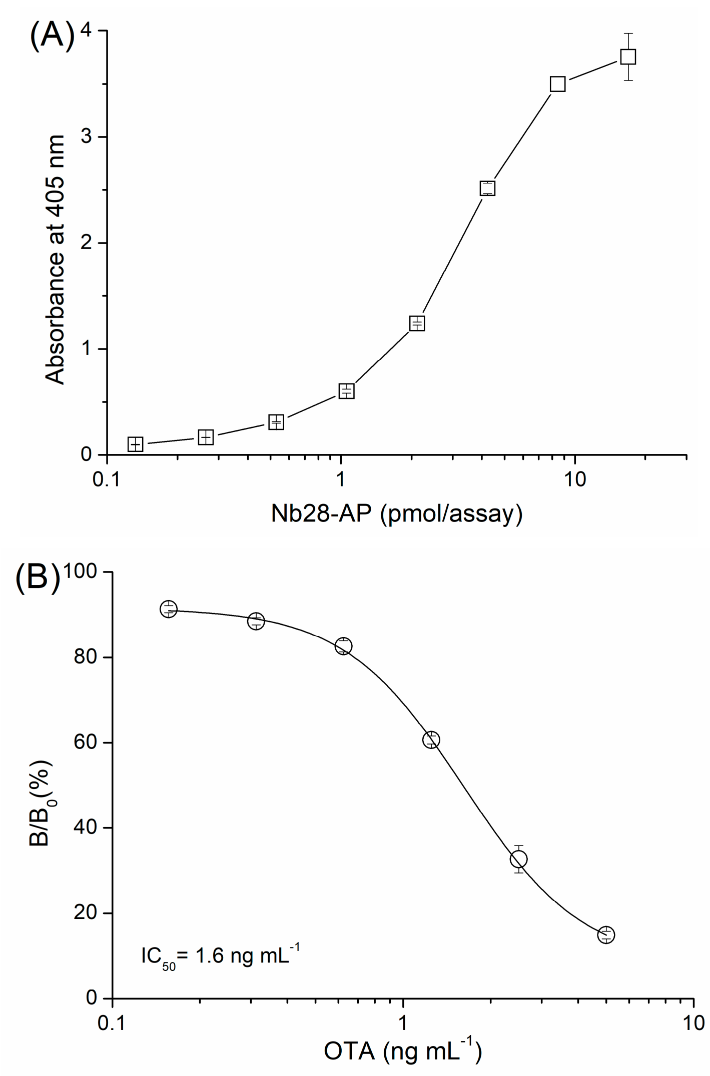

Figure S1. AP enzymatic activity and anti-OTA reactivity analysis of Nb28-AP. (**A**) Dose-response curve for AP enzymatic activity of Nb28-AP by colorimetric analysis. (**B**) Indirect competitive inhibition curve using Nb28-AP by colorimetric analysis. The error bars represent the standard deviation of three independent experiments.

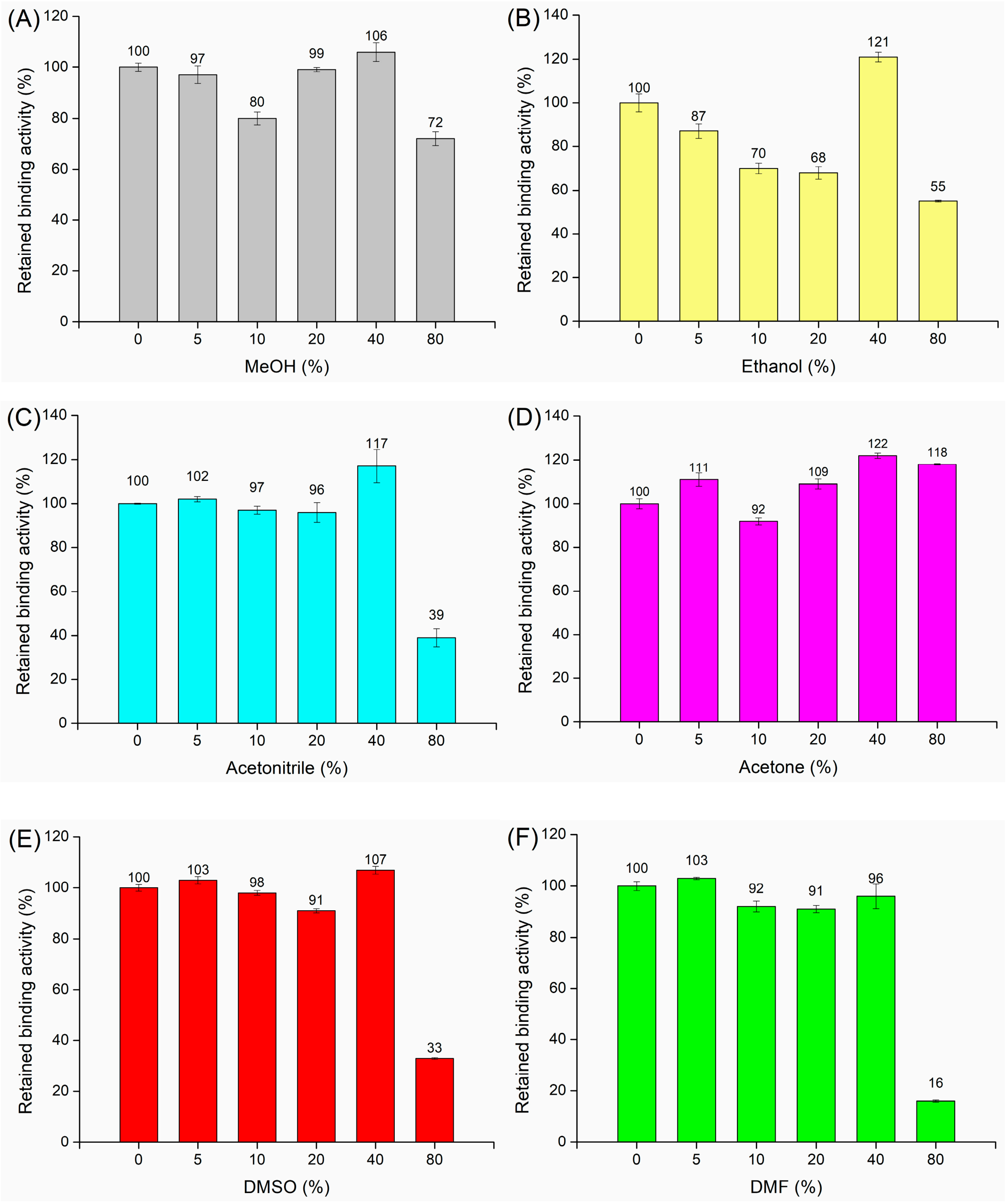

Figure S2. The solvent tolerance of Nb28-AP for methanol (**A**), ethanol (**B**), acetonitrile (**C**), acetone (**D**), DMSO (**E**), and DMF (**F**). PBS buffers containing each organic solvent at different concentrations (0%, 5%, 10%, 20%, 40%, and 80%) were used to dilute Nb28-AP, and 100 μL of the diluent was added into the wells coated with OTA-BSA. The bound Nb28-AP was detected by adding 100 μL of pNPP substrate solution. The error bars represent the standard deviation of three independent experiments.

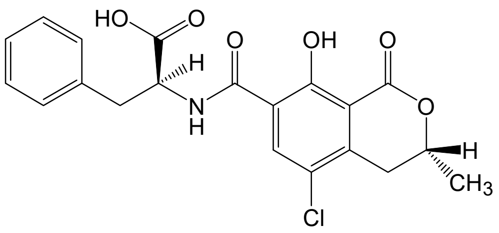

OTA

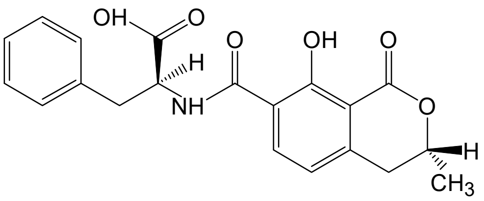

OTB

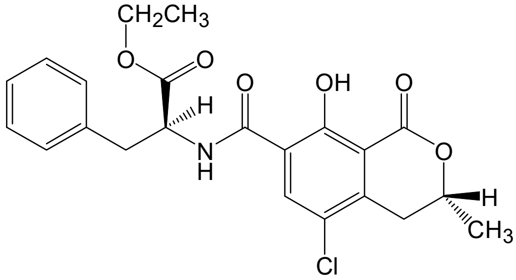

OTC

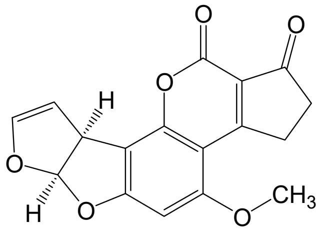

AFB$_1$

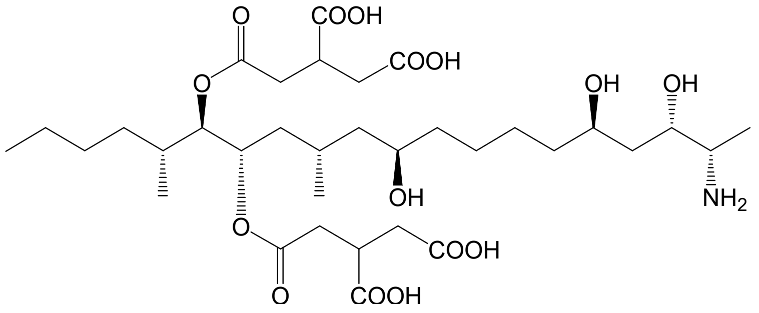

FB$_1$

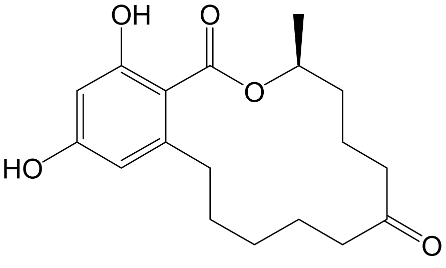

ZEN

Figure S3. The chemical structures of OTA, OTB, OTC, AFB$_1$, FB$_1$, and ZEN.

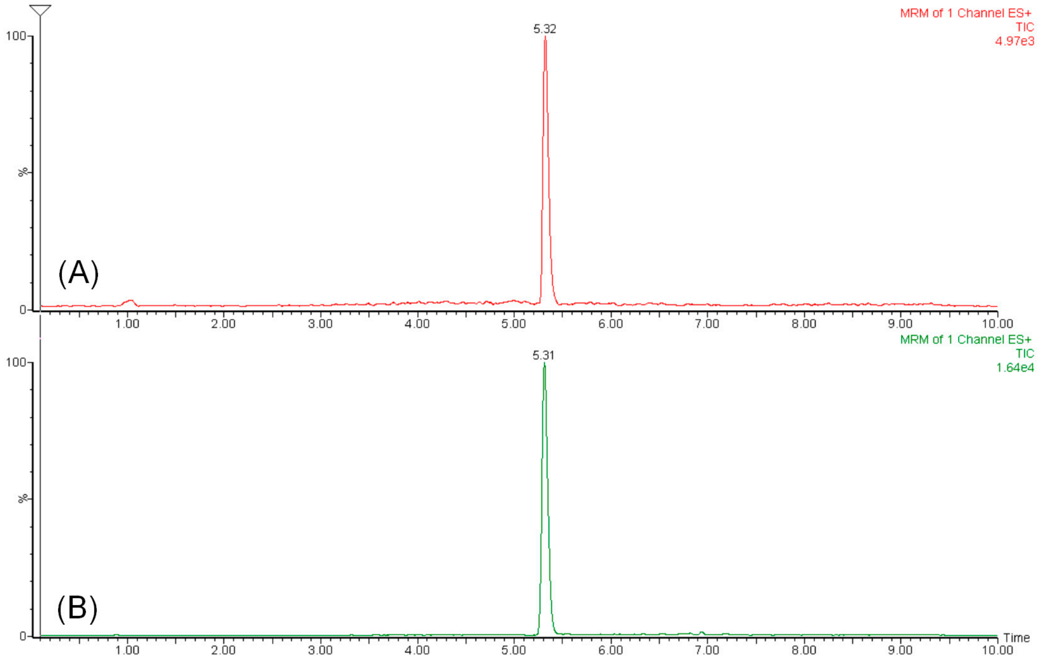

Figure S4. LC–MS/MS analysis of the OTA-contaminated rice sample (**A**) and the OTA standard (**B**).
